# Institutional Violence Against Users of the Family Law Courts and the Legal Harassment Scale

**DOI:** 10.3389/fpsyg.2019.00001

**Published:** 2019-01-18

**Authors:** Miguel Clemente, Dolores Padilla-Racero, Pablo Espinosa, Adela Reig-Botella, Manuel Gandoy-Crego

**Affiliations:** ^1^Unit Research in Criminology, Legal Psychology and Penal Justice, Department of Psychology, University of A Coruña, A Coruña, Spain; ^2^Department of Psychiatry, Radiology and Public Health, University of Santiago de Compostela, Santiago de Compostela, Spain

**Keywords:** family law, legal harassment, legal system, scale, violence

## Abstract

The term harassment is often used to refer two contexts, the workplace and school, but not the legal system itself. Long drawn-out litigation in the Family Law Courts often produces a surreptitious phenomenon of violence toward one of the litigating parties, who become victims of the legal system itself. The aim of this study was to determine whether legal harassment could be detected and measured in the Spanish Justice System using an innovative Legal Harassment Scale (LHS). This hypothesis was substantiated by the data obtained using a new 32-item psychometric instrument with a global index: the LHS, consisting of four factors: Direct Aggression, Procedural Harassment, Personal Contempt, and Manipulation of Reality. The estimated reliability and validity of the LHS was satisfactory, both in terms of the global score, and for each of the four factors distributed along the normal curve. The results of this study are discussed in terms of the limitations of the study and in relation to future lines of research aimed at ensuring that the legal system respects and safeguards the rights of the parties involved in litigation, and that no party falls victim to legal harassment.

## Introduction

What is legal harassment? Legal harassment or abuse was defined by Vollans (2010, p. 5) as follows: “Court-related abuse and harassment is the use of ongoing litigation through judicial and quasi-judicial systems to continue to harass and abuse." This abuse can affect all parents and is often focused on legal proceedings involving custody issues. We will start by highlighting some studies involving the legal system in general, and subsequently we will refer to the case of custody. In this paper we will use the terms “legal system" and “justice system" interchangeably.

Few studies have examined this context, yet many of the users of the justice system feel victimized by the system itself, either as plaintiff or defendant, though the effect of harassment is often more acute in the former. Unfortunately, to our knowledge, there are no studies on harassment in the legal system with the exception of a few studies that bear some relation and will be examined below. In all societies, there are disadvantaged groups, who are scorned both by society and the justice system (Athwal and Burnett, 2014). Members of specific alienated groups, primarily submerged in the underground economy, are “disappearing” due to low-level harassment both by society and the justice system itself, which either fails to act or, when it does, it is “low key,” leaving criminals to go unpunished (Athwal and Burnett, 2014).

According to Stotzer (2014) meta-analysis of 33 studies examining how the justice system treats its users, strikingly, the justice system stigmatizes both litigating parties (i.e., the plaintiff and the defendant). This study highlights that those who transgress the law suffer harassment, illegal detention, assault, and an overall lack of protection from the justice system because the perpetrators are the very agents of the justice system and law enforcement agencies. Similarly, victims also suffer harassment and discrimination by agents of the justice system and law enforcement agencies. In short, the law enforcement agencies and the justice system harass both victims and aggressors.

Moreover, the way in which victims are dealt with by the justice system affects their mental health. A study of 1562 United States Army reservists who had suffered sexual abuses in the army revealed that when they were treated fairly by the justice system and their statement was taken correctly and respectfully, the victim’s mental health improved, in particular, posttraumatic stress levels (Bell et al., 2014). Conversely, mental health declined in those individuals who felt they had been treated disrespectfully by the justice system. A study undertaken on a prison population found similar results (Smith, 2012). In short, we hypothesize that, in general, the mental health both of victims and aggressors improves when they feel they have been treated fairly and respectfully by the justice system. Conversely, we also hypothesize the inverse relationship on mental health occurs if victims feel they have been treated unfairly and disrespectfully.

A plausible explanation for this discrimination, from a legal point of view, can be found in the concept of “legal consciousness” (Silbey, 2005). In general, this theory draws a clear distinction between the theoretical concept of the law and its day-to-day functioning. Thus, the application of the law is claimed to be iatrogenic (i.e., individual application of the law often has adverse effects on victims). This author raised the issue of why people are willing to allow a legal system to exist that preaches equality, but systematically produces inequality.

In the area of mental health, the issue researched in the current study has is not been investigated (i.e., the types of harassment the justice system exerts on parents confronting each other in family court proceedings). We will now examine the case of legal harassment within family law procedures.

Our society has been recognizing different forms of violence, especially those perpetrated against women, with the current most frequent forms being mobbing, domestic violence, and sexual abuse (Epstein and Goodman, 2018). These authors also raised other social and cultural forms of acts against women, highlighting the lack of credibility granted to women’s reports. This is especially worrisome when we refer to the legal system, because when a woman reports that she or her children suffer abuse, she is much less likely to be believed than are men (Epstein and Goodman, 2018). We are thus faced with a paradox, because, on the one hand, laws are designed to protect women from abuse and, on the other hand, if women report abuse, they face a system which, being social, generates the defects found in society as a whole, and therefore it devalues their stories, often refusing to defend women with the laws created for this purpose (Epstein and Goodman, 2018).

This lack of credibility granted to women’s reports is manifest both in the legal system and in the social services derived thereof (Epstein and Goodman, 2018). Unfortunately, some fathers have even murdered the mothers and kidnapped and sometimes killed their children (Jaffee et al., 2003; Saunders, 2009; Rivera et al., 2012). It seems obvious that, faced with this situation, the courts must take extra measures to avoid serious consequences. The study of Rivera et al. (2012) shows that mediation can be a negative and revictimizing experience for women who have suffered abuse by their ex-partner. In a qualitative study carried out with 22 survivors of domestic violence who tried to negotiate safe children agreements after the separation, Laing (2016) reaches similar conclusions. The legal system’s attempt to support shared custody and mediate to achieve agreements between the parents at times clashes with the fathers’ accusations of the mothers’ Parental Alienation Syndrome (PAS), and their desire to conceal and silence domestic violence. This increases women’s secondary victimization. Therefore, mediation can be harmful for mistreated mothers and their children.

Regarding how the justice system becomes the harasser of women, abusers can use the justice system as a form of control of the other person and a form of harassment (Vollans, 2010). Thus, the court becomes an instrument for the abuser to be able to continue harassing the victim. But the fact that the abuser continuously forces the victim to litigate not only makes the victim relive a prior situation of abuse but it also attacks the victim economically. Both Vollans (2010) and Rivera et al. (2012) refer to the fact that many abusers are charismatic, charming and present very well in front of judges and experts, such that they convince these decision-makers that they (the abusers) are the real victims.

The report of Vollans (2010) proposed the goal of documenting the problem of abuse and harassment related to the courts. These authors cite a series of ways for abusers to use the justice system to attack and harass victims, which conform to the provisions of the law. This is one of the reasons why it is called an invisible form of abuse. This violence is invisible first because it occurs within intimate environments (home, for example), and secondly because society itself does not conceive it as such (for many people it is impossible to think that social institutions generate violence). As the system becomes a complicit in abusive behavior, the report by Vollans (2010) is very enlightening. This violence is invisible first because it occurs within intimate environments (home, for example), and secondly because society itself does not conceive it as such (for many people it is impossible to think that social institutions generate violence). The report by Vollans (2010) investigated the presence of 22 criteria that were selected for referring to court-related abuse or vexatious proceedings.

This harassment is sometimes achieved through the allegations of PAS, to which we will refer below. In this way, the family court becomes an invisible form of harassment.

The ways used to discredit mostly women’s testimony are primarily exhibited by the judges, because the judges ignore women’s reports of abuse and violence and consider them inconsistent, because they are not familiar with the symptoms derived from a traumatic situation, and especially those of a post-traumatic stress disorder (Epstein and Goodman, 2018). Judges and legal officers may not understand the severe mental health consequences that such traumatic situations produce. Thus, the judges and legal officers likely wonder if there are hidden motivations underlying the victims’ requests for help (Epstein and Goodman, 2018). In this way, some female victims also become revictimized by the legal system. In addition, the devaluation of their abuse experience and testimony makes victims wary to appeal to the justice system, which is supposed to protect them.

There is some evidence that family courts award the custody of minors to abusers, increasingly separate children from their mothers who wish to protect them, and these awards increase when the mother alleges child sexual abuse (Meier and Dickson, 2017). In addition, judges routinely do not recognize domestic violence or child abuse, they do not understand the consequences of abuse, and seek to maximize the fathers’ access to their children, whether or not they abuse their children (Meier and Dickson, 2017). It would be logical for the judge or the prosecutor to explore these allegations in order to determine whether the complaint is unfounded; however, in many cases, both the judges and the professionals who make up the teams that advise the legal system, entertain allegations of PAS (Meier and Dickson, 2017).

The study of Meier and Dickson (2017) on how PAS allegations affect the custody of fathers and mothers yields troubling data. In 72% of the cases in which PAS was legally claimed, the judge awarded custody to the father and removed the child(ren) from the mother. This percentage rose to 100% if the mother argued that the father was sexually abusing the children. It is also interesting, that if there was any suspicion of the mother’s mental impairment, in 50% of the cases, her custody is removed. Meier and Dickson (2017) conclude that the family courts manifest prejudice against women who report abuses by fathers; thus, family courts are potentially hostile places for mothers. Women run a significant risk of losing custody and the courts are especially punitive toward women and children who present complaints of sexual abuse.

All this is related to the meaning that judges grant to the concept of “the child’s best interests,” a question studied, among others, by Clemente et al. (2015), as well as by Naughton et al. (2015). In the case of this second group of authors, the analysis is performed according to the use of cognitive and heuristic schemas employed by judges. These authors argue that, like many people, judges idealize the concept of family unity, which itself is conceived as a nuclear family. The knowledge that there has been domestic violence goes against that idea and, in order to find coherence with the first idea, judges minimize, normalize, and trivialize such violence, considering its existence irrelevant. Thus, if a mother claims the existence of violence, she is assumed to be mentally ill or “crazy” for inventing a reality that does not exist. In general, therefore, these authors claim that the judges’ values act as a framework to explain their decision making.

The current study develops a new scale to measure a specific type of harassment, legal harassment, which injures parties involved in custody litigation. The victims, children, and parents, who struggle to defend the best interests of their children and to protect them from parent violence or sexual abuse. Thus, a new instrument was designed to measure legal harassment, and its impact on both of the litigating parties. The instrument has several factors, thus a further aim of this study was to estimate the reliability and validity and the factors.

## Materials and Methods

### Participants

An incidental sample was taken with the following inclusion criteria: all subjects were involved in ongoing litigation either as plaintiff, defendant, or both. Only litigations in the Family Law Courts were included in study, as they are long, drawn-out processes, especially litigations over child custody and visitation rights, which may be extended on until the child reaches the legal age of adulthood (18 years). Due to this circumstance, many of the subjects were both plaintiffs and defendants, although most were the latter. The sample consisted of 209 parents, 72.9% were women, with a mean age 40.28 years (range: 18–59), who had been involved in child custody litigation for more than 12 years (range: 2–12 years). The data were recollected between January and December 2017, in Galicia (NW Spain).

### Procedure

An *ad hoc* 78-item scale – called “the battery” – was designed, using situations related to harassment in the legal system. The initial items of the scale (the battery) were obtained with the collaboration of three experts, all of them psychologists who worked for the Justice System, and who had extensive experience in the follow-up of cases in which the litigant parties had been appealing to the court of justice for years. A factorial analysis was performed to reduce the number of items of the scale in order to design the Legal Harassment Scale (LHS).

The responses to the LHS were rated on a Likert-type scale, ranging from 0 (strongly disagree) to 4 (strongly agree). The application instructions were as follows:

“You have been involved in legal proceedings for some time. Read each of the statements below, indicate if the statements are applicable to you in your legal proceedings: I strongly disagree (0), I agree a little (1), I agree moderately (2), I agree a lot (3) I strongly agree (4). Thank you for cooperation.”

In addition, three extensively used tests were employed to determine the validity of the LHS:

- The 8-item Spanish version (Bobes et al., 2000) of the Top 8 Scale of Davidson and Colket (1997), which measures the frequency and severity of the symptoms of the posttraumatic stress disorder. Responses are rated on a 5-point Likert-type scale ranging from 0 (not at all frequent) to 4 (extremely serious).
- The Maslach Burnout Inventory, MBI (Maslach and Jackson, 1986), consisting of 30 items measuring three dimensions (Emotional fatigue, Depersonalization, and Personal accomplishment), and a global index estimate. Responses are rated on a 5-point Likert-type scale ranging from 0 (never) to 4 (every day).
- The Symptom Checklist of Derogatis SCL-90-R (Derogatis and Cleary, 1977a,b), consisting of 90 items measuring the following dimensions: Somatization, Obsession-Compulsion, Interpersonal sensitivity, Depression, Anxiety, Hostility, Phobic anxiety, Paranoid ideation, and Psychoticism. This scale also calculates global psychosomatization indices. The adapted Spanish version (Derogatis, 2001) was used (see Derogatis et al., 1976; Derogatis and Cleary, 1977a,b). Responses are rated on a 5-point Likert-type scale ranging from 0 (not at all) to 4 (very much or extremely).

In this work, we shall refer to harassment in the sense of a term in which psychological violence is exerted against people. Thus, we excluded physical violence. We consider that it is necessary to investigate the violence that the justice system directs against its users, as this is the main idea of this work.

Each participant completed the 78-item self-administered battery, plus all the tests mentioned above. Each test was performed individually. Therefore, data collection was extended for one year. Participants who completed the test were contacted through lawyers who acted as intermediaries. We think that the sample is of great interest, and given the mentioned difficulties, fairly large. Participants who informed their lawyers that they would participate were contacted by phone, and a visit was scheduled, if possible at the University, or if not, at their home. All signed an informed consent. The questionnaires were self-administered; the surveyor only clarified doubts and made sure that all the questions were answered.

After gathering the participants’ responses to the initial 78 scale items, factorial analysis was performed using principal components analysis of the correlation matrix (eigenvalues higher than 1), and varimax rotation (maximum variance, such that the first factor yields a higher loading than the second one, and so on successively). Scale reliability was tested using Cronbach’s alpha coefficient; concurrent validity was determined with the Pearson correlation with the three aforementioned tests; kurtosis and asymmetry were calculated by determining the fit on the normal curve.

All the information was gathered anonymously. At no time were the participants requested to give any identification data.

Approval for this study was requested from the Ethics Committee of the Universidade da Coruña (Spain) and obtained (Protocol No. 32/17). All the participants were informed about the objective of the investigation and, before completing the questionnaires, they signed an informed consent. In the consent form, they were ensured of the confidentiality and anonymity of their data. They were also requested to set a personal code so that, should they decide to cancel their consent within 2 months, their data could be deleted. However, no one refused to participate, and all signed the consent.

The IBM SPSS (Statistical Package for Social Sciences), version 22.0, was used for data analysis. Previously, the data were recorded in Excel, and the data of the questionnaires were confirmed.

## Results

### Analysis of Statistical Properties of the Legal Harassment Scale (LHS)

The Kaiser-Meyer-Olkin (KMO) measure of sampling adequacy was 0.941. Bartlett’s sphericity test was also calculated, obtaining a chi-square of 5820.670 (df = 496, *p* = 0.001). As the KMO value is very close to 1, the relation between variables is very high; that is, the test is very good. Regarding Bartlett’s sphericity test, as the value is <0.05, the null hypothesis was accepted; that is, factor analysis can be applied.

Firstly, an exploratory factor analysis was performed, finding that only four factors had an eigenvalue greater than 1, and they explained a minimum of 5% of the variance. Next, confirmatory factor analysis was conducted, specifying four factors, which was the test incorporated in the manuscript.

The results obtained revealed four factors. The first factor explained 20.626% of the variance, the second one explained 18.013%, the third one explained 15.280%, and the fourth one accounted for 13.589% after rotation (before rotation: 44.888, 13.845, 5.255, and 3.521%, respectively). That is, taken together, the four factors explained 67.508% of the variance.

Table 1 shows the factorial loadings of each item on each factor. Items are listed according to the size of the loadings. The eight items with the highest loadings on each factor were considered representative. An item was only included on a factor if it had a loading higher than 0.40, and was pure (only loading above 0.40 on one factor).

**Table 1:** Factorial analysis after rotation solution of the Legal Harassment Scale.

Statements	F I: 20.626	F II: 18.013	F III: 15.280	F IV: 13.589
1. I get written threats or telephone calls to my house	0.926			
2. I get verbal threats or intimidating gestures	0.917			
3. They try to hurt me physically to intimidate me	0.837			
4. They make indecent and cruel jokes about me	0.789			
5. I get verbally insulted	0.775			
6. They try to alienate me from my family and friends	0.739			
7. My accuser treats me as if I were mentally ill or implies I am	0.711			
8. They damage my home and/or my place of work	0.708			
9. The judge and/or lawyers no longer address me directly		0.825		
10. In general, I am legally ignored, and my version of the facts are ignored		0.813		
11. They do not give me the chance to speak		0.700		
12. When I make any legal applications to the courts, they normally refuse my requests or hinder me with drawbacks		0.679		
13. They do not give me the chance to explain anything, when I begin to say anything, they cut me off by asking me questions		0.677		
14. The judge and/or the prosecution interrupt me when I am speaking and do not let me finish what I want to say		0.665		
15. I get legally attacked without any consideration		0.618		
16. Negative confidential reports are issued about me, without being notified or given the opportunity to defend my self		0.607		
17. They ask me very specific questions to make me nervous and frustrated so I contradict myself			0.742	
18. I am forced to discuss things that make me nervous			0.725	
19. They try to put me under pressure by asking a barrage of questions			0.701	
20. I feel defenseless against their arguments			0.651	
21. I am forced to give very personal information			0.644	
22. They force me into litigation so I incur legal fees and expenses in order to harm me			0.620	
23. They put pressure on me by revealing intimate personal details			0.588	
24. I am forced to respond to absurd questions			0.578	
25. They underplay or belittle my efforts, achievements, successes, and merits				0.756
26. They disregard my skills and abilities				0.743
27. They exaggerate my faults and errors				0.700
28. My actions are under strict supervision				0.655
29. They maliciously distort everything I say				0.556
30. My decisions are always undermined or challenged				0.517
31. I get ferocious and unjust criticism or am mocked about aspects of my private life				0.491
32. They provoke me so I react emotionally				0.474


The analysis of the items of each factor determined the name assigned to the factor. Thus, the first factor, Direct Aggression, refers to harassment suffered through direct aggression, which normally occurring outside the courtroom such as in family settings and/or the workplace. The second factor, Procedural Harassment, refers to acts of harassment during legal proceedings, in particular in the courtroom by ridiculing victims under cross-examination and their testimonies. The third factor, Personal Contempt, refers to harassment or contempt through omission, for instance, ignoring a victim. The fourth and last factor, Manipulation of Reality, encompassed items concerning the disregard or undermining of the victim’s positive aspects while exaggerating negative aspects.

The items were randomized for subsequent presentation to other subjects. The randomized scale is shown in Supplementary Appendix 1. A column indicates the factor to which each item belongs. Table 2 presents the fit of the global legal harassment score and of the four factors to the normal curve, as well as the percentages.

**Table 2:** Scores for the Legal Harassment Scale.

Percentage	Average score Factor I (direct aggression)	Average score Factor II (procedural harassment)	Average score Factor III (personal contempt)	Average score Factor IV (manipulation of reality)	Average score global scale
5	0.000	0.000	0.000	0.000	0.137
10	0.000	0.125	0.250	0.375	0.312
15	0.125	0.125	0.625	0.625	0.481
20	0.125	0.250	0.750	0.875	0.656
25	0.250	0.250	0.875	1.000	0.843
30	0.375	0.375	1.000	1.125	0.968
35	0.500	0.393	1.125	1.268	1.093
40	0.625	0.625	1.325	1.500	1.218
45	0.750	0.750	1.381	1.750	1.312
50	0.875	0.875	1.625	2.000	1.406
55	1.125	1.000	1.750	2.125	1.625
60	1.375	1.250	2.000	2.375	1.718
65	1.525	1.481	2.125	2.500	1.787
70	1.875	1.500	2.250	2.625	1.987
75	2.250	1.625	2.375	2.750	2.062
80	2.500	1.775	2.500	3.000	2.187
85	2.625	2.000	2.956	3.375	2.425
90	2.900	2.125	3.125	3.500	2.843
95	3.625	2.750	3.500	3.750	3.087
99	3.875	3.625	4.000	4.000	3.531


The four factors were confirmed to be independent by examining the values of the covariances, which were practically zero, indicating that, in effect, the factors did not correlate with each other. The statistics of each factor and of the global scale were as follows: Direct Aggression, ranged between 0.00 and 3.88, arithmetic mean of 1.26, and standard deviation of 1.14; Procedural Harassment ranged between 0.00 and 3.63, arithmetic mean of 1.05, and standard deviation of 0.87; Personal Contempt ranged between 0.00 and 4.00 points, arithmetic mean of 1.68, and standard deviation of 1.03; Manipulation of Reality ranged between 0.00 and 4.00 points, arithmetic mean of 1.93, and standard deviation of 1.13; and the Global Scale ranged between 0.00 and 3.53 points, arithmetic mean of 1.48, and standard deviation of 0.87.

### Reliability, Validity, and the Fit to Normality of the New Scale

The reliability of the proposed scale was calculated using Cronbach’s alpha coefficient, both globally and for each of the factors. The results indicated high reliability, with the highest consistency being observed in the global scale (0.960), and the lowest in Factor II (0.896), which, nonetheless, almost reached 0.90. Factors I (0.939), III (0.918), and IV (0.936) obtained high reliability. Thus, the global scale and its factors were highly reliable. Intra-class correlation coefficients were calculated, reaching a value of 0.429 for individual measurements, and of 0.960 for the mean measurements. The *F*-test with true value 0 (25.24, both for individual measurements and for mean measurements) was highly significant (*p* < 0.001).

We also calculated composite reliability for each of the scale factors. Specifically, we calculated the KMO indices of each scale, as well as the Chi-square values. For the Direct Aggression scale, the KMO index value was 0.92, and the chi square was 1400.08 (df = 28, *p* = 0.000). For the Procedural Harassment scale, the KMO index value was 0.89, and the chi square was 782.92 (df = 28, *p* = 0.000). For the Personal Contempt scale, the KMO index value was 0.91, and the chi square was 783.38 (df = 28, *p* = 0.000). And for the Manipulation of Reality scale, the KMO index value was 0.91, and the chi square was 1055.25 (df = 28, *p* = 0.000). In the corresponding factor matrix of each factor, the items loadings of Factor I ranged between 0.922 and 0.686, with a mean of 0.811; the items of Factor II ranged between 0.836 and 0.601, with a mean of 0.705; the items of Factor III ranged between 0.907 and 0.590, with a mean of 0.763; and the items of Factor IV ranged between 0.927 and 0.580, with a mean of 0.775.

To determine the concurrent validity of the scale, Pearson correlations were calculated between the overall LHS scores and its four factors with the scores of the SCL-90-R, the TOP-8, and the MBI. The LHS showed significant positive correlations of 0.01 or higher with all the global scores of these tests. High scores on the LHS correlated significantly with high levels of psychosomatization, posttraumatic stress, and burnout. Thus, the LHS can be considered to show satisfactory concurrent validity.

Correlations were also determined for each of the LHS factors with the SCL-90, MBI, and TOP-8 factors (Table 3). The correlations for between Direct Aggression and the global scores of all of the scales were highly significant. In comparison, Procedural Harassment only correlated significantly with the global MBI score, and two of its subscales: Emotional fatigue and Personal accomplishment. Similarly, Personal Contempt only correlated significantly with the global MBI score, and three of its subscales. As for Manipulation of Reality, similar to Factor I, it correlated significantly with all of the global scores of the other questionnaires, and with many of the subscales. In short, all of the factors correlated significantly with burnout, and Direct Aggression and Manipulation of Reality also did so with psychosomatic symptomatology and posttraumatic stress.

**Table 3:** Pearson’s correlations between LHS and other tests.

	Factor I: direct aggression	Factor II: procedural harassment	Factor III: personal contempt	Factor IV: manipulation of reality	Overall score
Somatization (SCL-90-R)	0.27**			0.14*	0.16*
Obsession-Compulsion (SCL-90-R)	0.20**			0.16*	0.16*
Interpersonal sensitivity (SCL-90-R)	0.23**			0.17*	0.17*
Depression (SCL-90-R)	0.18**			0.17*	0.15*
Anxiety (SCL-90-R)	0.28**			0.17*	0.20**
Hostility (SCL-90-R)	0.31**			0.16*	0.20**
Phobic anxiety (SCL-90-R)	0.21**				
Paranoid ideation (SCL-90-R)	0.26**	0.14*	0.14*	0.27**	0.26**
Psychoticism (SCL-90-R)	0.17*				
Global SCL-90-R	0.25**			0.18*	0.19**
Emotional fatigue (MBI)	0.39**	0.20**	0.28**	0.35**	0.38**
Depersonalization (MBI)	0.38**		0.15*	0.29**	0.29**
Personal accomplishment (MBI)	0.37**	0.20**	0.26**	0.34**	0.37**
Global burnout	0.40**	0.19**	0.27**	0.36**	0.38**
TOP-8	0.34**			0.25**	0.24**


*^∗∗^Correlation is significant at 0.01 (bilateral). ^∗^Correlation is significant at 0.05 (bilateral).*

Finally, the normality of the scale and of the four factors was determined. Asymmetry and kurtosis values showed adequate normality.

Hence, it was possible to measure legal harassment, and to obtain a global index as well as an index for each of its four components. Thus, the scale fulfills the relevant statistical requirements.

## Discussion and Conclusion

This work creates a scale of legal harassment, which is produced within the procedures of family law. This scale is made up of four factors: Direct Aggression, Procedural Harassment, Personal Contempt, and Manipulation of Reality. Direct Aggression refers to actions that take place outside of the legal realm, usually within the area of their families and their work, involving attacks on justice system users. Procedural Harassment is similar to the former, but it takes place within the courtroom or during the various legal proceedings (i.e., interrogations, statements, etc.), such that the person who has become a victim is ridiculed. Personal Contempt refers to actions in which the victim is ignored, despised, or treated with contempt. Finally, Manipulation of Reality refers to the fact that the victim’s abusers (judges, prosecutors, and lawyers of the opposing party) present a distorted image of the victim, exaggerating or even inventing negative aspects, and concealing or misrepresenting the positive aspects, turning them into negative ones. In this way, the victim is completely stigmatized, and the institution allows and encourages this mistreatment, which we have termed institutional harassment.

We draw from the idea that harassment can be produced by the legal system as a whole. The judge, insofar as he or she must direct and order the process, is a part of the legal system. Normally the judge has the power to limit or allow the prosecutor and the counsel of the opposing party to harass one or both of the parties. For this reason, the scale we propose not only refers to the actions of harassment exerted by judges, but by all the parties involved in the legal procedure of custody.

This scale allows us not only to measure the degree of institutional harassment exercised by the legal system on its users, but also to determine the extent to which it is higher or lower in each of the four specified components.

Given the high correlation between the LHS scores, which was created with variables that have been regularly used to determine people’s mental health – and specifically the existence of posttraumatic stress – it can be concluded that legal harassment, besides being another form of harassment, negatively affects people’s mental health. To be attacked within a legal process implies an attack on the mental health of those who are harassed from within the system.

The most similar work to our line of research is the meta-analysis that has examined how people are treated within the justice system (Stotzer, 2014). However, it did not deal with the issue researched herein, that is, the harassment the justice system exerts on parents confronting each other in family court proceedings.

We are aware of the limitations of this work, some identified below. The main problem of this paper is the sample. Firstly, because it is incidental, and secondly because its size is quite limited. It is necessary to point out that, although the size of our sample may seem small, as it was not possible to examine more than one subject at a time, the difficulties to get participants in a legal family process are considerable. It was very difficult to gain access to the people who formed it, and no legal body was willing to facilitate access to the litigants. Therefore, we had to resort to the family lawyers, who acted as intermediaries so that we could contact their clients and request their participation. No doubt, if the administration of justice would collaborate in the future, it would be feasible to use larger samples, and hopefully not incidental ones. Not in vain, the consultation of previous works shows that many of the investigations that have been carried out use a qualitative methodology, or they resort to sentences or news, instead of interviewing the people involved in the process.

The majority of the members of the sample are women. We believe that this is commonplace in family processes research, in which there is a greater response rate in the group of women. However, for future work, we will attempt to obtain samples with a higher number of males.

On the basis of the arguments specified above, the type of legal proceedings is family law. At least, in Spanish law, family processes, and therefore custody, are settled in two courts, the so-called Family Courts and Courts of Violence against Women. We believe that in the future, it would be interesting to use this distinction, but in both cases, violence and custody issues are treated.

We want to stress that in a subject like this, two sides of the same coin need to be considered. Thus, future work should be aimed at the study of both victims and aggressors, and perpetration and victimization experiences of both should be measured. We again note that the aggressor population is difficult to access. Therefore, research designs and strategies that allow obtaining data from both victims and aggressors on the subject should be used.

We believe it is very important to work on the creation of protocols that prevent the legal harassment of all parties by imposing ethical guidelines for the way lawyers question the opposite party, in the way other legal professionals address parties, and the behavior of the judges and prosecutors (Vollans, 2010; Rivera et al., 2012; Naughton et al., 2015; Laing, 2016; Meier and Dickson, 2017; Epstein and Goodman, 2018). The formation of protocols for the treatment parties in court proceedings is necessary and urgent. The search for the truth of the facts and the delivery of justice should not be incompatible with fair and respectful treatment of parties.

It is necessary to take into consideration that we are addressing issues of family law where there are children of a couple that has separated. When these parents come to the justice system in such circumstances, it is because the parents have failed to agree, or have been unwilling to do so. Revenge can be a motivator for the parties and should be measured in future work.

Until now, judges have been considered to be responsible for issuing fair decisions, but their organization of the process of oral hearings and of treatment of users in the process has received little attention. Not taking this issue into account can lead to serious injustice toward users of the legal system, and even to mistreating them, especially in family law.

## Compliance with Ethical Standards

The procedures performed in this study were in accordance with the ethical standards of the institutional Ethic Committee of the University of A Coruña, Spain (ref. 32/17) and with the 1964 Helsinki declaration and its later amendments or comparable ethical standards. Informed consent was obtained from all individual participants included in the study.

## Author Contributions

All authors have contributed equally to the development of this research and to the elaboration of the manuscript. MC, DP-R, and PE specially have participated in the design and analysis of the data. MG-C and AR-B specially have participated in the elaboration of the report. All authors have participated in the preparation of psychological tests, in the elaboration of the data matrix, in the elaboration of conclusions and discussion, and agreed the final version of this manuscript.

## Conflict of Interest Statement

The authors declare that the research was conducted in the absence of any commercial or financial relationships that could be construed as a potential conflict of interest.
