# Supplementary material for: Institutional Violence Against Users of the Family Law Courts and the Legal Harassment Scale
Source: Front Psychol. 2019 Jan 18;10:1. doi: 10.3389/fpsyg.2019.00001 (PMC6345693; doi:10.3389/fpsyg.2019.00001)
Supplement: Supplementary file 1 [file Data_Sheet_1.docx]

Appendix 1: The Legal Harassment Scale

| You have been involved in legal proceedings for some time. Read each of the statements below, indicate if the statements are applicable to you in your legal proceedings using the following scale: I strongly disagree (0), I agree a little (1), I agree moderately (2), I agree a lot (3) I strongly agree (4). Thank you for cooperation. | **FACTOR** | **I strongly disagree 0** | **I agree a little 1** | **I agree moderately 2** | **I agree a lot 3** | **I strongly agree 4** |
| --- | --- | --- | --- | --- | --- | --- |
| 1. I am forced to respond to absurd questions | III | 0 | 1 | 2 | 3 | 4 |
| 1. I am forced to give very personal information | III | 0 | 1 | 2 | 3 | 4 |
| 1. They try to hurt me physically to intimidate me | I | 0 | 1 | 2 | 3 | 4 |
| 1. They provoke me so I react emotionally | IV | 0 | 1 | 2 | 3 | 4 |
| 1. I get verbal threats or intimidating gestures | I | 0 | 1 | 2 | 3 | 4 |
| 1. They do not give me the chance to speak | II | 0 | 1 | 2 | 3 | 4 |
| 1. They disregard my skills and abilities | IV | 0 | 1 | 2 | 3 | 4 |
| 1. They maliciously distort everything I say | IV | 0 | 1 | 2 | 3 | 4 |
| 1. My actions are under strict supervision | IV | 0 | 1 | 2 | 3 | 4 |
| 1. They make indecent and cruel jokes about me | I | 0 | 1 | 2 | 3 | 4 |
| 1. They damage my home and/or my place of work | I | 0 | 1 | 2 | 3 | 4 |
| 1. My decisions are always undermined or challenged | IV | 0 | 1 | 2 | 3 | 4 |
| 1. I get ferocious and unjust criticism or am mocked about aspects of my private life | IV | 0 | 1 | 2 | 3 | 4 |
| 1. They underplay or belittle my efforts, achievements, successes, and merits | IV | 0 | 1 | 2 | 3 | 4 |
| 1. I get written threats or telephone calls to my house | I | 0 | 1 | 2 | 3 | 4 |
| 1. I get legally attacked without any consideration | II | 0 | 1 | 2 | 3 | 4 |
| 1. They force me into litigation so I incur legal fees and expenses in order to harm me | III | 0 | 1 | 2 | 3 | 4 |
| 1. When I make any legal applications to the courts, they normally refuse my requests or hinder me with drawbacks | II | 0 | 1 | 2 | 3 | 4 |
| 1. They try to alienate me from my family and friends | I | 0 | 1 | 2 | 3 | 4 |
| 1. They try to put me under pressure by asking a barrage of questions | III | 0 | 1 | 2 | 3 | 4 |
| 1. My accuser treats me as if I were mentally ill or implies that I am | I | 0 | 1 | 2 | 3 | 4 |
| 1. Negative confidential reports are issued about me, without being notified or given the opportunity to defend my self | II | 0 | 1 | 2 | 3 | 4 |
| 1. I get verbally insulted | I | 0 | 1 | 2 | 3 | 4 |
| 1. They exaggerate my faults and errors | IV | 0 | 1 | 2 | 3 | 4 |
| 1. In general, I am legally ignored, and my version of the facts are ignored | II | 0 | 1 | 2 | 3 | 4 |
| 1. They ask me very specific questions to make me nervous and frustrated so I contradict myself | III | 0 | 1 | 2 | 3 | 4 |
| 1. The judge and/or lawyers no longer address me directly | II | 0 | 1 | 2 | 3 | 4 |
| 1. They do not give me the chance to explain anything, when I begin to say anything, they cut me off by asking me questions | II | 0 | 1 | 2 | 3 | 4 |
| 1. I´m forced to discuss things that make me nervous | III | 0 | 1 | 2 | 3 | 4 |
| 1. The judge and/or the prosecution interrupt me when I am speaking and do not let me finish what I want to say | II | 0 | 1 | 2 | 3 | 4 |
| 1. They put pressure on me by revealing intimate personal details | III | 0 | 1 | 2 | 3 | 4 |
| 1. I feel defenceless against their arguments | III | 0 | 1 | 2 | 3 | 4 |
